# The Combined Effects of High-Intensity Interval Exercise Training and Dietary Supplementation on Reduction of Body Fat in Adults with Overweight and Obesity: A Systematic Review

**DOI:** 10.3390/nu16030355

**Published:** 2024-01-25

**Authors:** Eliza Gaweł, Barbara Hall, Szymon Siatkowski, Agata Grabowska, Anna Zwierzchowska

**Affiliations:** 1 Institute of Sport Sciences, The Jerzy Kukuczka Academy of Physical Education in Katowice, Mikolowska Street 72a, 40-065 Katowice, Poland; e.gawel@awf.katowice.pl (E.G.); a.zwierzchowska@awf.katowice.pl (A.Z.); 2 Institute of Healthy Living, The Jerzy Kukuczka Academy of Physical Education, Mikolowska Street 72, 40-065 Katowice, Poland; s.siatkowski@awf.katowice.pl; 3 Faculty of Medical Sciences in Katowice, Medical University of Silesia in Katowice, 40-055 Katowice, Poland; d201159@365.sum.edu.pl

**Keywords:** body fat, high-intensity interval exercise, dietary supplementation

## Abstract

Excessive body fat is associated with various comorbidities including cardiovascular disease, type 2 diabetes mellitus and certain types of cancer. The search for effective, relatively easy to maintain body-fat reduction interventions has been ongoing. We aimed to review the current literature to assess the effectiveness of high-intensity interval training with and without dietary supplementation on body fat loss, concentration of markers of metabolic health and aerobic capacity of adults with overweight and obesity. Seventy full-text articles were assessed to determine their eligibility and thirteen were included in the review. The methodology of this systematic review was developed according to the Preferred Reporting Items for Systematic Reviews and Meta-Analyses (PRISMA) guidelines. Almost all studies (93%) demonstrated effectiveness of high-intensity interval training of various protocols in reducing body fat, improving metabolic health and aerobic capacity of adults with overweight and obesity. These effects were enhanced by an addition of a dietary supplement, such as green tea or ginger or other. Although combining HIIT with dietary supplementation seem to improve body composition, metabolic health and aerobic capacity in adults with overweight and obesity in some instances to a greater extent than HIIT alone, it does not seem to be necessary to combine these two interventions.

## 1. Introduction

The prevalence of overweight (BMI ≥ 25 kg/m^2^) and obesity (BMI ≥ 30 kg/m^2^) has been dramatically increasing since the last century and these disorders are now affecting 59% of adults and almost 1 in 3 children in Europe [1,2]. Worldwide, approximately 2 billion adults are overweight and of these around 650 million are obese [3]. It is expected that 2.7 billion adults will be overweight and over 1 billion will be obese by 2025 [4]. 

An abnormal or excessive body fat (BF) accumulation is associated with impaired cellular metabolism [1] and various comorbidities. These include cardiovascular disease [5], type 2 diabetes mellitus [6], non-alcoholic fatty liver disease [6], certain types of cancer [5] and mental disorders [7]. The BF accumulation and its related comorbidities involve complex pathogenesis, including adipokines dysregulation (pro-inflammatory > anti-inflammatory profile) [8] and inhibition of browning of the white adipose tissue, which leads to reduced dissipation and increased storage of chemical energy [9]. Microbiota dysbiosis characterised by a depletion of probiotics that may increase the risk of colorectal cancer [10] and immune dysregulation with immune cells shifting from the anti-inflammatory to pro-inflammatory phenotype are other hallmarks of BF accumulation [8,11].

A sedentary lifestyle and reduced physical activity in conjunction with unhealthy hypercaloric diet [12], rich in glycaemic (digestible) carbohydrates (CHO) such as sugar and refined CHO [13,14] and fat [14] and genetic, endocrine, metabolic and environmental factors are viewed as the main causes of the excessive BF [15]. Reducing BF is generally achievable for a person with overweight or obesity; however, maintaining the reduced BF level has been proven to be challenging and only a very small percentage of these individuals succeed in maintaining the results [15]. Therefore, the quest for finding effective and relatively easy to adhere to and to maintain therapeutic interventions has been ongoing.

Since compliance with standard body mass management programmes is notoriously poor [16], various dietary strategies are recommended to decrease BF and amongst these are dietary supplements (DS). DS for BF management can be classified into herbs and botanicals, vitamins, minerals, and amino acids [17]. A recent excellent systematic review has demonstrated that most DS as aids in reducing BF have a limited, high-quality evidence-based efficacy [18]. For instance, out of twenty-two studies on calcium-vitamin D-supplementation, only three demonstrated significant body mass reduction over time and of the five papers with low bias (23%), none showed significant changes in body mass between groups (experimental vs. placebo). Out of sixteen studies on green tea supplementation with an overall low degree of bias, only two (13%) demonstrated statistically significant improvements in %BF ranging from 1.7 to 2.3%, with no statistical differences between treatment and placebo groups. On the other hand, ephedrine–caffeine (often taken together) [18] supplementation seemed to be more effective as of the ten studies with a low risk of bias, 50% reported significant BF reduction ranging from 0.3 to 4.9 kg but results on inter-group pre/post changes were not reported. The authors concluded that well-designed, randomised, double-blinded controlled trials of sufficient duration are needed to show efficacy of DS in BF loss and suggested that adding DS to lifestyle interventions to enhance efficacy would be helpful to explore [18].

High-intensity interval exercise training (HIIT) has been gaining popularity for its time-saving characteristics, being perceived as more enjoyable and giving a greater sense of accomplishment when compared to traditional moderate intensity exercise training (MICT) [19]. HIIT can be defined as a near-maximal (intensity of 85–95% of maximal heart rate), or maximal or supramaximal, bouts of between ≤ 30 s and 6 min of work, interspersed with 2–5 min of rest [20]. HIIT has been shown to improve a number of cardiovascular and metabolic risks factors in healthy, older and non-obese females [21], sedentary males with overweight [22], patients with coronary artery disease [23] and patients with type 1 diabetes [24]. There is growing evidence that HIIT elicits greater benefits than MICT on a range of health markers in the healthy and those suffering from chronic illnesses [25,26]. A meta-analysis of the effectiveness of HIIT and MICT on BF loss demonstrated that both regimens could induce only modest body composition improvements in individuals with overweight or obesity [27]. However, some studies in people with obesity revealed that HIIT can significantly reduce BF [28,29]. Without the consensus to whether HIIT regimens are effective for BF loss and whether the BF loss can be maintained, other therapeutic interventions such as dietary supplementation (DS) has been used in combination with HIIT to assess their effectiveness in BF reduction and markers of metabolic health.

To the best of the authors’ knowledge, to date, no study has analysed the synthesis of the results of HIIT combined with DS in adults with overweight and obesity. Therefore, the aim of the study was to examine the effectiveness of HIIT combined with DS on BF loss, markers of metabolic health and aerobic capacity in adults with overweight and obesity.

## 2. Materials and Methods

### 2.1. Study Design

The methodology of this systematic review was developed according to the Preferred Reporting Items for Systematic Reviews and Meta-Analyses (PRISMA) guidelines (Prisma—internet) (see Supplementary Materials File S1).

### 2.2. Inclusion and Exclusion Criteria

In this systematic review, the inclusion criteria for the study were as follows: (1) randomised controlled trials (RCTs) (including double-blind RCTs), (2) males and females 18–50 years old, (3) individuals with overweight/obesity diagnosed based on the WHO guidelines (BMI ≥ 24.99 kg/m^2^) [1,2], (4) no other health conditions, (5) HIIT combined with DS.

The exclusion criteria were: (1) a study design different than RCT (2) a study group including children, adolescents or people aged over 50 years old, (3) a poor methodological design, (4) no full text available, (5) manuscript written in a language other than English, (6) lack of data, (7) HIIT not combined with DS or DS not combined with HIIT.

### 2.3. Literature Search 

A search of the selected electronic databases (Pubmed, SCOPUS, ScienceDirect) was conducted by two authors (SS, AG). They identified all studies on HIIT combined with DS and their effects on BF in individuals with overweight and obesity conducted between November 2013 and November 2023. The following methods were used during the search: (a) data mining, and (b) data discovery and classification. The search terms were combined by Boolean logic (AND/OR) in PubMed, SCOPUS and ScienceDirect databases. The search was undertaken using the following 7 prioritised keyword combinations in English: ‘HIIT’, ‘adiposity, ‘supplementation, ‘overweight’, ‘obesity’, ‘adipose tissue’, ‘body fat’ and ‘reduction’. Furthermore, two other authors (EG, BH) with expertise in somatic analysis, overweight and obesity, HIIT and DS reviewed the reference lists of the included studies and screened Google Scholar for additional studies. The corresponding authors of the selected publications were also contacted directly if the crucial data were not available in the original articles.

### 2.4. Methodological Quality of the Included Studies (Risk of Bias)

The methodological quality of the included studies was evaluated based on a revised Cochrane risk of bias tool—RoB 2.0 tool (version of 22 August 2019) that is known to be suitable for individually-randomised, parallel-group and cluster RCT [30]. RoB 2.0 tool is also known to be the most recommended risk of bias tool for RCT [30]. The tool consist of five bias domains: (1) risk of bias arising from the randomisation process, (2) risk of bias due to deviations from the intended interventions (effect of assignment interventions/adhering the intervention), (3) missing outcome data, (4) risk of bias in measurement of the outcome and (5) risk of bias in the selection of the reported results, that is followed by an ‘overall risk of bias’ assessment [31]. Each study was read and ranked by two independent investigators (EG, SS) with one of the following response options: (1) ‘yes’ (2) ‘probably yes’, (3) ‘probably no’, (4) ‘no’, (5) ‘no information’, (6) ‘no applicable’ to a series of signalling questions in each of the five domains. Moreover, an independent co-author (AZ) was designated to resolve all discrepancies that could occur among investigators during the assessment. Based on the original RoB 2.00 tool, the responses that were underlined in green were believed to have a low risk of bias, while the responses underlined in red were believed to have a high risk of bias [31]. Next, based on the obtained responses, each investigator (in each of the domains) chose one of the following risk of bias judgment options, i.e., (i) ‘low’, (ii) ‘high’ or (iii) ‘some concerns’ [31]. According to the recommendations for the overall risk of bias judgement, if all domains were judged as ‘low’ risk of bias, the overall risk of bias was low, while if one domain was judged as a ‘high’ risk of bias, or if several domains were judged to have ‘some concerns’, the overall risk of bias was high. Moreover, if at least one domain was judged as ‘some concerns’, but simultaneously there was no domain judged as ‘high’ risk of bias, the overall risk of bias was judged to have some concerns [31]. Furthermore, based on the original recommendations from Sterne et al. [31], RoB 2.0 tool was used to assess the risk of bias based on a given outcome, rather than for a whole RCT.

## 3. Results

### 3.1. Study Selection

**Figure 1:**
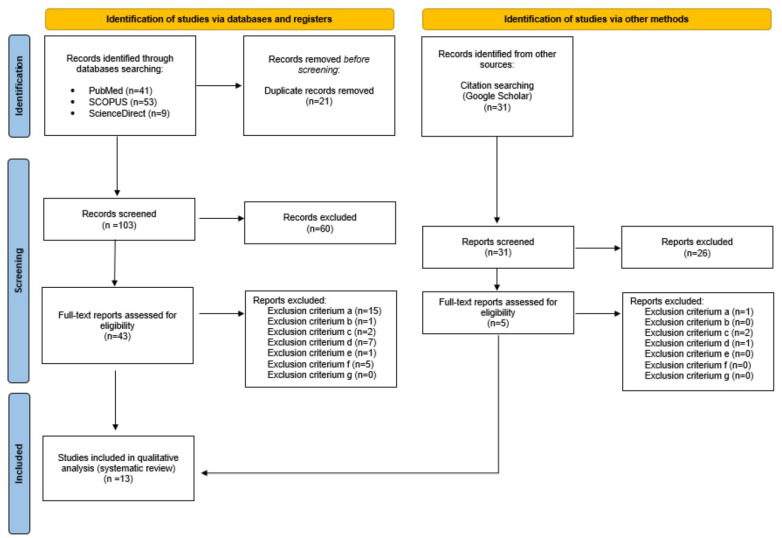
PRISMA flow diagram detailing the study inclusion process (PRISMA—internet).

The flow of the systematic review is presented in Figure 1. Seventy full-text articles were assessed to determine their eligibility, while fifteen met the inclusion criteria and were subjected to a detailed analysis and assessment of their methodological quality (see Table 1).

The majority of the reports that were assessed for their methodological quality were considered to have a low overall risk of bias and were judged eligible to be included in the systematic review, while some of the publications were considered to have some concerns of eligibility and none of the reports were judged with a high risk of bias. The initial agreement of the two independent investigators (EG, BH) was 90%. All discrepancies among the investigators were resolved by an expert evaluation by an independent co-author (AZ). Thirteen full-text articles were finally included in the systematic review (see Table 2).

### 3.2. Study Characteristics

**Figure 2:**
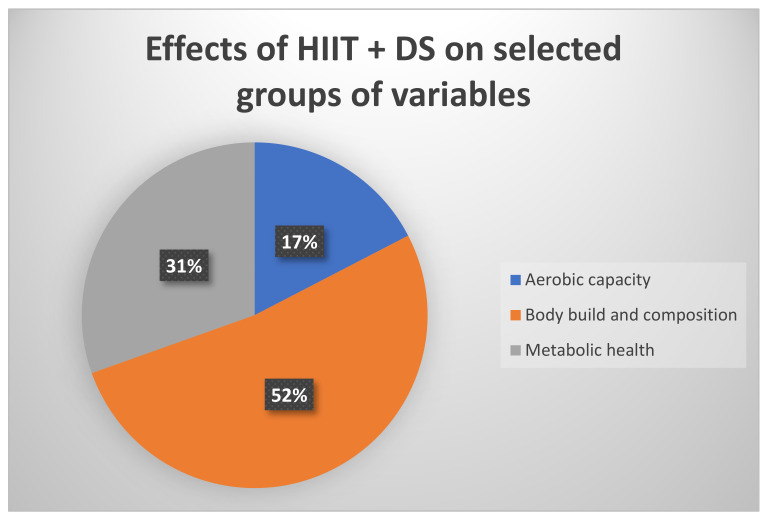
Effects of HIIT and DS (%) on selected groups of analysed variables, i.e., aerobic capacity, body build and composition, metabolic health.

A comparison of the effects of HIIT combined with DS on the selected variables analysed in the reviewed articles (see Table 1) is presented in Figure 2. It was found that body build and composition (52%) were the variables most frequently impacted by the interventions.

## 4. Discussion

A careful examination of the current scientific studies on the HIIT combined with DS on BF loss, aerobic fitness, and selected indices of metabolic health in adults with overweight and obesity yielded partially inconsistent findings. Nevertheless, this qualitative analysis found that HIIT combined with adequate DS can induce BF loss, reduce or increase blood concentration of metabolic health markers and improve aerobic capacity to a greater extent rather than DS or HIIT alone (see Table 2) in adults with overweight and obesity.

### 4.1. Effects of HIIT Combined with Supplementation on BF

The majority of the analysed scientific articles demonstrated statistically significant effects of HIIT and DS on BF and induced a reduction of %FAT, body fat mass, BMI, or waist-to-hip ratio [32,33,36,39,40,41,42,43,44]. On the contrary, two investigations [35,37] did not find any effects of HIIT combined with DS on BF in older adults with overweight and obesity. These inconsistencies could be explained mainly by the differences in the (1) HIIT protocol, (2) type, daily dose and duration of DS and (3) characteristics of the participants (sex, age, anthropometric measurements).

#### 4.1.1. HIIT: The Most Popular Protocol

Among different HIIT protocols, the 40-m shuttle run test was implemented most frequently [33,34,36,38,41] and was combined with ginger (3000 mg/daily) [33,34] or green tea supplementation [36,38], which proved to be effective in reducing BF of participants with overweight and obesity to a greater extent than HIIT or DS alone [33,36,38]. Conversely, Sheikholeslami-Vatani et al. [41] found that the 40-m shuttle run protocol combined with vitamin D_3_ (2000 IU/daily) or placebo supplementation both had a similar effect on the indicators of body fat loss: %FAT, BM and BMI, whereas Saghebjoo et al. [37] demonstrated that the 40-m shuttle run HIIT combined with L-arginine (6 g/daily) did not cause a significant reduction of the BF mass. The above-mentioned synthesis of the results allowed us to conclude that a 40-m shuttle run test as a HIIT protocol may be effective in inducing BF loss both alone and in combination with DS, especially with green tea.

#### 4.1.2. HIIT: Other Protocols

Apart from the 40-m shuttle run, other HIIT protocols had been implemented in the reviewed studies. Some of them used longer HIIT sessions, lasting between 30 and 60 min [42,43,44], whilst others focused on much shorter sessions [32,40] lasting between 8 and 30 s. Regardless of its duration, each HIIT session combined with DS (see Table 2) has been found effective in improving the body composition of participants with overweight and obesity, especially in reducing their BF. Regarding the duration of HIIT, shorter (6-week) and longer (up to 12 week) HIIT programmes were as effective in reducing BF in individuals with overweight and obesity.

#### 4.1.3. DS: Type and Dose Effect on BF

However, the effectiveness of a combined HIIT + DS intervention on body composition improvement seemed to depend on the type and dose of the DS. For instance, Lithgow et al. [35] demonstrated that a 6-week HIIT alone significantly reduced the waist and hip circumference of adults with overweight and obesity, with vitamin D_3_ (100 μg/day = 4000 IU/day) not proffering any additional effects. Similar observations were made by Sheikholeslami-Vatani et al. [41] who implemented a two-times lower dose of vitamin D_3_ (2000 IU/day) supplementation but a longer by two weeks intervention compared to Lithgow et al. [35]. The authors observed that both HIIT + vitamin D_3_ and HIIT + placebo reduced %FAT and BMI significantly (*p* < 0.05) and to a similar extent in males with overweight.

#### 4.1.4. HIIT + DS: Sex-Based Differences in BF Responses

Minor sex-based differences in the intrinsic responses to HIIT and DS were observed. Hirsch et al. [39] demonstrated that compared to females, males tended to have greater muscular adaptations (i.e., thigh muscle size and muscle quality) to HIIT alone and HIIT combined with essential-amino acid DS compared to females. In females, HIIT + EEA induced greater improvements in the muscle mass than HIIT alone, which suggests that EAA may support greater increases in muscle mass, especially in females. The sex-based dissimilarities could be attributed to hormonal differences between males and females, especially in the secretion of anabolic hormones and growth factors that induce muscle mass grow and in the skeletal muscle fibre-type composition [45] that could contribute to the body’s biochemical responses to HIIT and EAA supplementation.

Unfortunately, the currently available scientific literature that has examined the effects of HIIT combined with DS did not provide enough data to indicate the best supplement for a reduction of BF mass. Nevertheless, based on the detailed examination of the current scientific studies a direct effect of HIIT combined with several supplements on the improvement of body composition, especially BF reduction can be confirmed (see Table 2).

### 4.2. Effects of HIIT Combined with Supplementation on Metabolic Markers

The reviewed studies have analysed various markers of metabolic health in participants with overweight and obesity. In the current study, the effects of HIIT + DS on the most relevant metabolic markers are being discussed.

#### 4.2.1. Adipokines and Insulin

Adipokines are cytokines secreted by white and brown adipocytes that signal the functional status of these cells to targets in the muscle, blood vessels, liver, pancreas, brain and other tissues [46]. Adipokines exert numerous functions, including regulation of carbohydrate and lipid metabolism, appetite, immune functions (pro-and anti-inflammatory adipokines), development and maintenance of muscle mass, and many others [47,48]. Enhanced expression of pro-inflammatory cytokines in obesity plays a role in inducing insulin resistance [49], the state characterised by impaired insulin-stimulated glucose uptake by myocytes and adipocytes, with reduced inhibition of liver glucose production [50]. Consequently, elevated fasting glucose levels and the inability to effectively clear glucose from the circulation during the post-prandial state will occur [51], eventually leading to type 2 diabetes characterised by chronic hyperglycaemia and hyperinsulinaemia [52]. Adipokine dysregulation is observed in obesity and contributes to obesity-related disorders [46]. In the current review, five studies [32,37,42,43,44] assessed the effects of HIIT + DS on blood adipokines and insulin homeostasis and four of them found beneficial modifications [32,42,43,44]. Whereas the effects of HIIT alone or in combination with DS on fasting glucose and insulin, as the indicators of insulin resistance, without the effects of HIIT + DS on adipokines were assessed by two studies [35,41], which, interestingly, had chosen similar HIIT protocols and vitamin D_3_ supplementation.

##### Adipokines

Dunn et al. [32] demonstrated that a 12-week intervention consisting of 8-s sprint on a manual cycle ergometer + 12-s rest, 3 × a week combined with the ω-3 fatty-acids supplementation and a low-glycaemic diet, significantly reduced serum IL-6. This is an important finding as IL-6 is a pro-inflammatory cytokine that plays a role in insulin resistance [53]. The authors also observed a tendency for adiponectin to rise in response to the intervention. In contrast to IL-6, higher levels of adiponectin, an insulin-sensitising cytokine, are associated with augmented glucose and lipid metabolism preventing hyperinsulinaemia [46]. Indeed, this observation was accompanied by a significant reduction in fasting insulin and a tendency for improved insulin resistance index (HOMA IR), suggesting increased sensitivity of the insulin-target cells and better metabolic health. Conversely, Saghebjoo et al. [37] did not observe any significant effects of HIIT (30-s sprint, repeated 4–6 times depending on the week of HIIT) + L-arginine supplementation on serum adiponectin in males with overweight.

Leptin is one of the key adipokines produced by white fat cells and is concerned with suppression of appetite, hence, energy homeostasis, and inflammation [46,54]. Its serum concentration positively correlates with BF mass, and leptin resistance is another feature of obesity and type 2 diabetes [55]. In the current review, only one study assessed the effects of HIIT + DS on leptin [44]. The authors found that HIIT consisting of 30 min exercise sessions combined with citrulline (an amino acid) significantly reduced serum leptin in older adults with obesity and HIIT+ citrulline-induced effects were more profound compared to HIIT alone [44]. Reduction of leptin in response to HIIT + citrulline was accompanied by a significant reduction in the total android BF mass and a tendency for increased adiponectin, the results not observed in response to HIIT alone.

In their two studies, Saeidi et al. [42,43] observed beneficial effects of a 12-week high-intensity functional training Cross-fit^®^, a form of HIIT [56], combined with either astaxanthin [42] or spinach-derived thylakoid [43] on selected markers of metabolic health. Cross-fit^®^ + astaxanthin significantly reduced CTRP2, CTRP9, myostatin and growth differentiation factor-15 (GDF15) [42]. In obesity, these adipokines have been shown to be elevated and to negatively affect lipid and glucose metabolism, and skeletal muscle homeostasis [57,58,59,60]. Whereas Cross-fit^®^ + spinach-derived thylakoid decreased the proinflammatory leptin and resistin and increased the anti-inflammatory adiponectin and omentin in males with obesity, suggesting a reduction of inflammation, a feature of BF accumulation [8,11]. These findings were accompanied by reduced insulin resistance [42,43] and the beneficial metabolic effects were greater in the combined intervention demonstrating a synergistic effect of HIIT and astaxanthin/spinach-derived thylakoid.

##### Insulin

Lithgow et al. [35] demonstrated that HIIT (cycling for 1 min × 10 repetitions, 3 × week for 6 weeks) significantly reduced fasting insulin and glucose plasma levels, improving insulin sensitivity in males and females with overweight and obesity, whilst, interestingly, the addition of vitamin D_3_ appeared to attenuate the HIIT-induced improvement in glucose tolerance.

Sheikholeslami-Vatani et al. [41] revealed reduced fasting insulin, but not fasting glucose, in response to HIIT of a similar protocol [35], but a slightly longer duration of the investigation and a lower dose of vitamin D_3_ supplementation (2000 IU/day vs. 4000 IU/day compared to Lithgow et al. [31]. Similarly, vitamin D_3_ supplementation did not proffer any additional positive effects on glucose tolerance during an oral glucose tolerance test or fasting insulin levels suggesting a superior role of HIIT in achieving such improvements.

#### 4.2.2. Aerobic Capacity

Overweight and obesity are associated with reduced aerobic capacity and exercise tolerance which will negatively affect daily energy expenditure associated with physical activity and/or exercise and will further augment BF accumulation [61]. Therefore, improvements in aerobic capacity are of significant health importance and indicate improvement in the cardio-respiratory systems [62]. In the present review, nearly a half of studies assessed the effects of HIIT with or without DS on VO_2_max of adults with overweight and obesity. Maximal oxygen uptake (VO_2_max or VO_2_peak in patients with reduced exercise tolerance) is considered the golden standard of cardiopulmonary and muscle cell fitness [62]. VO_2_max increased in response to HIIT alone or combined with ω-3 fatty acids and low-glycaemic diet [32], green tea [38], ginger [33,34], astaxanthin [42] and spinach-derived thylakoid [43]. Ghasemi et al. [38] also found a significant elevation of PGC-1α, a key regulator of mitochondrial biogenesis in muscle, in response to HIIT + green tea, suggesting an improvement of cellular aerobic metabolism [63]. Apart from one [33], all studies demonstrated greater improvements in VO_2_max when HIIT was combined with DS.

It is worth noting that the type, dose and duration of dietary supplementation as well as participants’ characteristics, including sex, age and anthropometric indices could be responsible for the variability of the findings of the reviewed studies. In order to fully understand the complexity of interactions that occur between the intrinsic biochemical processes further studies are needed, including meta-analysis, to evaluate the effects of HIIT and DS on the reduction of BF, improvement of metabolic markers and aerobic capacity of adults with overweight and obesity.

## 5. Limitations and Strengths

While this qualitative analysis contributes to the current body of literature, there are some limitations that need to be addressed. The main limitation of the current study is a small number of studies that have investigated HIIT combined with DS in population of people with overweight and obesity, which did not allow for conducting a quantitative analysis that would allow for general interference. Moreover, the analysed studies were conducted with various number of participants including age and sex differences. Moreover, HIIT protocols differed in duration. In addition, various types and doses of dietary supplements make generalisation impossible. Therefore, further research is needed to fully understand the complexity of DS combined with HIIT that should be focused on delivering knowledge on an adequate type and dose of DS for people with overweight and obesity, that can be applied for healthy reduction of the adipose tissue. Moreover, there is still a need for studies to develop a universal model of HIIT protocol that can be combined with DS and applied to individuals with overweight and obesity. The main strength of the present study is the systematic review of the latest reports from the last decade that have examined the HIIT combined with DS in adults with overweight and obesity (similar age range). Moreover, the majority of the included studies were evaluated to be perfectly eligible for this analysis. The authors believe that the novelty of the presented research problem and undertaking the hitherto unexplored aspects in scientific research will enable a better understanding of the process of implementing HIIT in this population. It may also help to optimise HIIT programmes by using interventions based on sex-based strengths combined with specific DS.

## 6. Conclusions

- In general, HIIT combined with DS seems to induce the reduction of BF mass and improve metabolic health in adults with overweight and obesity; however, its effectiveness is related to several variables, including sex, type and dose of DS and HIIT protocol;
- The 40-m shuttle run test could be recommended as an effective form of HIIT for targeting overweight and obesity;
- It is difficult to point out the best type of DS for the reduction of BF in population with overweight and obesity; however, green tea seems to be effective.
- HIIT seems to be effective in reducing insulin resistance and does not seem to require being combined with DS;
- HIIT combined with DS green tea, ginger, astaxanthin or spinach-derived thylakoid may be more effective in improving aerobic capacity of adults with overweight and obesity than HIIT alone;
- Although combining HIIT with dietary supplementation seems to improve body composition, metabolic health and aerobic capacity in adults with overweight and obesity to a greater extent than HIIT alone, but it does not seem to be necessary to combine these two interventions.

## 7. Practical Implications

Individuals with overweight and obesity can face different barriers to exercise, including lack of time, feelings of negative body image and lack of confidence in exercising in front of others [64]. Therefore, HIIT with its time-consuming characteristics and its adaptability to a person’s level of exercise tolerance, may be a good solution for BF loss, improvement of metabolic health and aerobic capacity intervention. Some of these effects (i.e., profile of adipokines and aerobic capacity) may be enhanced by the addition of DS.

For instance, HIIT could be implemented into a daily routine by running instead of walking to a bus stop or up the stairs, skipping, hopping, performing jumping jacks at home or during a break at work, or interspersing moderate-intensity exercise such as jogging, swimming or cycling with intense, short bouts of the exercise. DS, such as green tea could be consumed prior to a HIIT session to enhance its BF lowering effects, potentially improve the profile of adipokines and aerobic capacity. Although, it was not within the scope of the current work, it would also be interesting to evaluate whether adding green tea, ginger, spinach or astaxanthin-rich algae, yeast, salmon, trout, krill, shrimp or crayfish [65].

## Figures and Tables

**Table 1:** The assessment of the methodological quality of the included studies (risk of bias) using the Cochrane RoB 2.0 tool (version of 22 August 2019) for RCT. Studies are presented in order of the publication year.

Author	Risk of Bias Judgement					
	Domain 1	Domain 2	Domain 3	Domain 4	Domain 5	Overall Risk of Bias
Dunn et al. [32]	LOW	LOW	LOW	LOW	LOW	LOW
Nayebifar et al. [33]	LOW	SOME CONCERNS	LOW	LOW	LOW	SOME CONCERNS
Lithgow et al. [34]	LOW	LOW	LOW	LOW	LOW	LOW
Afzalpour et al. [35]	LOW	SOME CONCERNS	LOW	LOW	LOW	SOME CONCERNS
Ghasemi et al. [36]	SOME CONCERNS	SOME CONCERNS	LOW	LOW	LOW	SOME CONCERNS
Saghebjoo et al. [37]	LOW	LOW	LOW	LOW	LOW	LOW
Ghasemi et al. [38]	LOW	LOW	LOW	LOW	LOW	LOW
Hirsch et al. [39]	LOW	LOW	LOW	LOW	LOW	LOW
Nobari et al. [40]	LOW	SOME CONCERNS	LOW	LOW	LOW	SOME CONCERNS
Sheikholeslami-Vatani et al. [41]	LOW	LOW	LOW	LOW	LOW	LOW
Saeidi et al. [42]	LOW	SOME CONCERNS	LOW	LOW	LOW	SOME CONCERNS
Saeidi et al. [43]	LOW	LOW	LOW	LOW	LOW	LOW
Youssef et al. [44]	LOW	LOW	LOW	LOW	LOW	LOW

**Table 2:** The summary of the studies from 2014 to 2023 evaluating the effects of HIIT and supplementation on reduction of body fat in individuals with overweight and obesity. Presented based on DS classification [17].

Author	Participants Characteristics	HIIT	Supplement	Protocol of the Intervention (HIIT + Supplement)	Intervention: Main Findings
Herbs and botanicals					
Nayebifar et al. [33]	nF = 24/age = 20–30 years SG1 (HIIT + ginger); nF = 8/age = 21.88 ± 3.4 years; BM = 74.19 ± 11.53 kg; BMI = 28.68 ± 2.60 kg/m^2^ SG2 (HIIT + placebo); nF = 8/age = 22.38 ± 3.24 years; BM = 72.24 ± 6.86 kg; BMI = 28.42 ± 2.4 kg/m^2^ SG3 (ginger); nF = 8/age = 21.63 ± 1.77 years; BM = 64.91 ± 3.6 kg; BMI = 26.06 ± 1.69 kg/m^2^	A 40 m shuttle run: three times per week, 20 m of max speed/20 m recovery, repeated 10 times.	Ginger (3000 mg) or placebo (Nokhodchi flour) 30 min before each meal.	A 10-week programme of HIIT combined with 3000 mg of ginger or Nokhodchi flour supplementation in females with overweight.	HIIT+ ginger: ↓ body fat (%) ∆ ↑ VO_2_max (mL/kg/min). HIIT: ↑ VO_2_max (mL/kg/min).
Afzalpour et al. [34]	nP = 24 females SG1 (HIIT + ginger); nF = 8/age = 21.87 ± 3.39 years; BM = 74.19 ± 11.53 kg; BMI = 28.68 ± 2.60 kg/m^2^ SG2 (HIIT + placebo); nF = 8/age = 22.37 ± 3.24 years; BM = 72.24 ± 6.86 kg; BMI = 28.42 ± 2.40 kg/m^2^ SG3 (ginger); nF = 8/age = 21.62 ± 1.76 years; BM = 64.91 ± 3.6 kg; BMI = 26.06 ± 1.6 kg/m^2^	A 30 s sprint (intensity over 90% HRmax) followed by 30 s active rest, and repeated three times per training session, three times/week. Each two weeks, one repetition was added.	Ginger (3 g per one tablet) or placebo (flour) once a day.	A 10-week programme of HIIT combined with ginger or placebo supplementation in females with overweight and obesity.	HIIT + ginger ∆: ↓ body fat (%), ↑ VO_2_max (mL/kg/min).
Ghasemi et al. [36]	nP = 30 females/age = 20–30 years; BMI ≥ 25 kg/m^2^ SG1 (green tea); nF = 10 SG2 (HIIT + green tea); nF = 10 SG3 (HIIT + placebo); nF = 10	A 40-m maximal shuttle run (30 s) at an intensity of 85–95% of maximum heart rate interspersed with 30 s of active relaxation time, three times a week for 10 weeks.	Green tea (500 mg) or placebo (500 mg starch powder) three times/day.	A 10-week programme of HIIT combined with 500 mg green tea extract or placebo supplementation in females with overweight.	HIIT + green tea ∆: ↓ body fat (%).
Ghasemi et al. [38]	nP = 30 females/age = 20–30; BMI > 25 kg/m^2^ SG1 (HIIT + green tea); nF = 10/age = 22.47 ± 3.32 years, BM = 70.56 ± 6.19 kg; BMI = 27.15 ± 1.47 kg/m^2^ SG2 (HIIT + placebo); nF = 10/age = 23.58 ± 2.23; BM = 72.18 ± 3.51 kg; BMI = 27.32 ± 1.27 kg/m^2^ SG3 (green tea); nF = 10/age = 21.06 ± 2.65; BM = 73.45 ± 8.44 kg; BMI = 28.03 ± 1.04 kg/m^2^	A 40-m shuttle run at 90% Hrmax (30 s) three times/week.	Green tea (1500 mg) or placebo (starch powder) tablets three times/day, 2 h after the main meals, 7 days/week. For 10 weeks.	A 10-week programme of HIIT combined with green tea (500 mg) supplementation in young, sedentary females with overweight.	HIIT + green tea ∆: ↓ body fat (%), ↓ body mass (kg), ↑ PGC-1α (pg/mL), ↑ VO_2_max (ml/kg/min).
Nobari et al. [40]	nP = 30 females/age = 25.1 ± 6.7 years; BM = 75.8 ± 8.4 kg SG1 (spirulina); nF = 10 SG2 (HIIT + spirulina); nF = 10 SG3 (HIIT + placebo); nF = 10	An exercise of 30 s running at an intensity of 90% Hrmax, interspersed with 30 s walking, repeated 4–7 times in each session, 3 times a week.	Spirulina powder (6 g) or placebo (green colouring food dissolved in water) one tablet per day.	An 8-week programme of HIIT and spirulina powder (6 g/day) or placebo supplementation in females with overweight and obesity.	HIIT + spirulina: ↓ body mass (kg)
Saeidi et al. [42]	nP = 68/age = 27.6 ± 8.4 years; BM = 94.7 ± 2.0 kg; BMI = 33.6 ± 1.4 kg/m^2^ SG1 (astaxanthin); nP = 15 SG2 (HIIT); nP = 15 SG3 (HIIT + astaxanthin); nP = 15 CG; nP = 15	Crossfit sessions of 60 min consisting of 60 min/CrossFit^®^ sessions consisting of squats, deadlift, press, jerks, barbell, dumbbell, and medicine ball cleans, pull-ups, kettlebell swings and other, three times a week.	Astaxanthin (20 mg) or placebo (20 mg raw corn starch) one tablet a day with breakfast.	A 12-week programme of 36 high-intensity-functional training Crossfit sessions lasting up to 60 combined with astaxanthin (20 mg/day) or raw corn starch supplementation in males with obesity.	HIIT + astaxanthin ∆: ↓ body fat (%), ↓ body mass (kg), ↓ BMI (kg/m^2^), ↓ fasting glucose (mg/dL), ↓ fasting insulin (ng/mL), ↓ myostatin, GDF-15, CTRP2 and CTRP9 (ng/mL), ↑ VO_2_peak (mL/kg/min).
Saeidi et al. [43]	nP = 68/age = 27.6 ± 8.4 years; BM = 95.7 ± 3.8 kg; BMI = 32.6 ± 2.6 kg/m^2^ SG1 (spinach-derived thylakoid); nP = 15 SG2 (HIIT); nP = 15 SG3 (HIIT + spinach-derived thylakoid); nP = 15 CG; nP = 15	Crossfit sessions of 60 min consisting of 60 min/CrossFit^®^ sessions consisting of squats, deadlift, press, jerks, barbell, dumbbell, and medicine ball cleans, pull-ups, kettlebell swings and other.	Spinach-derived thylakoid (5 g) or placebo (5 g of raw corn starch).	A 12-week programme of 36 high-intensity-functional training Crossfit sessions lasting up to 60 min combined with thylakoid-rich spinach extract or matching placebo supplementation.	HIIT + spinach-derived thylakoid ∆: ↓ body fat (%), ↓ HOMA IR, ↓ myostatin, GDF-15, CTRP2 and CTRP9 (ng/mL), ↑ fat free mass (kg), ↑ VO_2_peak (mL/kg/min).
Vitamins					
Lithgow et al. [35]	nP = 20, males and females SG1 (HIIT + vitamin D; nP = 10; nM = 8; nF = 2/age = 34 ± 9 years; BM = 96.2 ± 12.2 kg; BMI = 30.5 ± 2.2 kg/m^2^ SG2 (HIIT + placebo); nP = 10; nM = 6; nF = 4/age = 34 ± 10 years; BM = 97.0 ± 15.6 kg; BMI = 32.3 ± 3.1 kg/m^2^	A cycle ergometer: 10 repetitions of 1 min intervals interspersed with 1 min active recovery at a power output of 50, three times a week for 6 weeks. The power output (W) of the high-intensity intervals was assigned as a workload corresponding to 100% VO_2_peak (sessions 1–6) to 110% VO_2_peak (sessions 7–12), and ultimately 120% (sessions 13–18)	Vitamin D_3_ (100 μg per tablet) or placebo one tablet/day with breakfast.	A 6-week programme of HIIT combined with vitamin D or placebo supplementation in males and females with overweight and obesity.	HIIT alone: ↓ waist and hip circumference (cm) HIIT + vitamin D_3_: ↓ fasting insulin (mU/L) ↓ fasting glucose (mmol/L)
Sheikholeslami-Vatani et al. [41]	nP = 48 males/age = 21.7 ± 1.4 years; BM = 86.52 ± 3.92 kg; BMI = 27.28 ± 0.76 kg/m^2^ SG1 (HIIT + Vitamin D_3_); nM = 12 SG2 (HIIT + placebo); nM = 12 SG3 (Vitamin D_3_); nM = 12 CG; nM = 12	An amount of 10 × 1 min intervals cycling at 90% VO_2_peak separated by 1 min active recovery at 15% VO_2_peak from week 1 to week 4, then 10 × 1 min intervals cycling at 100% VO_2_peak separated by 1 min active recovery at 15% VO_2_peak from week 5 to week 8; 3 sessions per week.	Vitamin D_3_ (2000 IU) or placebo (maltodextrin) one tablet a day.	An 8-week programme of HIIT combined with vitamin D_3_ (2000 IU/day) or maltodextrin supplementation in males with overweight.	HIIT alone and HIIT + vitamin D_3_, ↓ body fat (%), ↓ BMI (kg/m^2^), ↓ body mass (kg), ↓ insulin (ng/dl).
Amino acids					
Saghebjoo et al. [37]	nP = 40 males/age = 23.9 ± 1.4; BMI = 29.6 ± 4.0 kg/m^2^ SG1 (HIIT + placebo); nM = 10 SG2 (L-arginine); nM = 10 SG3 (HIIT + L-arginine); nM10 SG4 (placebo); nM = 10	A 30-s sprint interspersed with 30 s of walking, repeated 4–6 times in the first and second week, to 5 times in the third and fourth week then 6 times in the fifth and sixth week.	L-arginine (6 g/day): three capsules per day with 400 mL of water at half an hour before breakfast, 1 h before lunch, and 1 h before the last daily meal.	A 6-week programme of HIIT combined with L-arginine (6 g/day) supplementation in males with overweight and obesity.	No significant changes in %FAT, BMI, adiponectin in response to HIIT, L-arginine or HIIT + L-arginine.
Hirsch et al. [39]	nP = 76 males and females SG1 (HIIT); nP = 19; nM = 9; nF = 10/age = 36.74 ± 5.61; BM = 96.57 ± 17.23 kg; BMI = 31.73 ± 4.72 kg/m^2^ SG2 (EAA); nP = 20; nM = 10; nF = 10/age = 35.60 ± 4.95; BM = 96.66 ± 16.33 kg; BMI = 31.22 ± 4.29 kg/m^2^ SG3 (HIIT + EAA); nP = 19; nM = 9; nF = 10/age = 36.21 ± 6.65; BM = 91.78 ± 13.54 kg; BMI = 31.41 ± 3.36 kg/m^2^CG; nP = 8; nM = 4; nF = 4/age = 36.88 ± 7.45; BM = 92.20 ± 15.52 kg; BMI = 30.55 ± 3.91 kg/m^2^	On a cycle ergometer: 1-min at 90% max wattage interspersed with 1-min complete rest. HIIT started with six sets of intervals and progressed by one set each week until reaching ten sets at week five; ten sets were maintained for the remainder of the 8 weeks; HIIT sets were performed 2 days/week.	Essential amino acid (EAA) (3.6 g) twice a day.	An 8-week programme of HIIT combined with EAA (3.6 g/2× day) supplementation in adults with overweight and obesity.	HIIT alone and HIIT+ EAA: ↑ thigh lean mass size (kg)
Youssef et al. [44]	nP = 83; BM = 81.4 ± 14.0 kg; BMI 30–40 kg/m^2^ SG1 (HIIT +citrulline); nP = 39 SG2 (HIIT + placebo); nP = 44	HIIT, three times/per week; 30 min/session.	Citrulline (10 g) or placebo daily.	A 12-week HIIT combined with citrulline or placebo supplementation in adults with obesity.	HIIT + citrulline: ↓ total body fat (kg and %), ↓ android and trunk fat mass (kg), ↓ leptin (ng/mL).
Fatty acids					
Dunn et al. [32]	SG; nF = 15/age = 24 ± 1.0 years; BM = 73.3 ± 3.1 kg; BMI = 27.6 ± 0.8 kg/m^2^ CG; nF = 15/age = 22 ± 0.6 years; BM = 70.9 ± 2.9 kg; BMI = 25.7 ± 0.5 kg/m^2^	A manual cycle ergometer, three times a week, 20 min of exercise: 8-s sprint, 12-s recovery.	ω-3 fatty acids (550 mg of eicosapentaenoic acid and docosahexaenoic acid per 1100 mg capsule) three capsules/day.	A 12-week programme of HIIT combined with low glycaemic Mediterranean diet (Mediet) and ω-3 supplementation in females with overweight.	HIIT+ ω-3 + Mediet: ↓ total fat mass (kg), ↓ abdominal adiposity (kg), ↓ waist circumference (cm), ↓ systolic blood pressure (mmHg), ↓ fasting plasma insulin (μIU/mL), ↓ IL-6 (pg/mL), ↖ VO_2_peak (ml/kg/min).

nP = number of participants; nF = number of females; nM = number of males; SG—study group; CG—control group; BM—body mass; BMI—body mass index; PGC-1α—peroxisome proliferator-activated receptor-gamma coactivator; GDF-15—growth differentiation factor-15, CTRP-2—C1q-TNF related protein 2; CTRP9—C1q-TNF related protein 9; IL-6—interleukin 6; HIIT—high intensity interval training; HR-heart rate; VO_2_max—maximal oxygen consumption; VO_2_peak—peak oxygen consumption; SD—standard deviation.; ↓ significant reduction; ↑ significant increase; ↖ tendency to increase; ∆—indicates more prominent improvement compared to HIIT or DS alone.

## Data Availability

The data presented in this study are available on request from the corresponding author. The data are not publicly available due to privacy reasons.

## Supplementary Materials

The following supporting information can be downloaded at: https://www.mdpi.com/article/10.3390/nu16030355/s1, File S1: PRISMA 2020 Checklist. Reference [66] is cited in the Supplementary Materials.
